# Extrarenal renal cell carcinoma in the adrenal region: a case report

**DOI:** 10.3389/fsurg.2024.1449879

**Published:** 2024-10-18

**Authors:** Kai Yao, Long Huang, Jing Li Zhang, Yan Xu, Dong Liang Liu

**Affiliations:** Department of Urology, 363 Hospital, Chengdu, China

**Keywords:** extrarenal renal cell carcinoma, adrenal region, case report, adrenal masses, clear cell renal cell carcinoma

## Abstract

This case report describes a rare instance of extrarenal clear cell renal cell carcinoma (ccRCC) in a 48-year-old woman who presented with a loss of consciousness. Abdominal CT revealed a 24 × 31 mm mass in the left adrenal region, with no kidney involvement. The mass was surgically excised, and histopathological examination confirmed the diagnosis of ccRCC. Immunohistochemical analysis revealed positive markers, including CA9, CD10, PAX-8, and vimentin. The patient did not undergo adjuvant therapy, and a 6-month follow-up showed no signs of recurrence or metastasis. This case emphasizes the importance of considering extrarenal ccRCC in differential diagnoses of adrenal masses.

## Introduction

Renal cell carcinoma (RCC) is a prevalent malignancy within the urinary system, with clear cell renal cell carcinoma (ccRCC) being the most common histological subtype, accounting for approximately 70% of RCC cases (1). Extrarenal RCC, however, is extremely rare, with only a few cases documented in the literature (2–4). In this report, we present a case of extrarenal ccRCC located in the adrenal region.

## Case report

**Figure 1:**
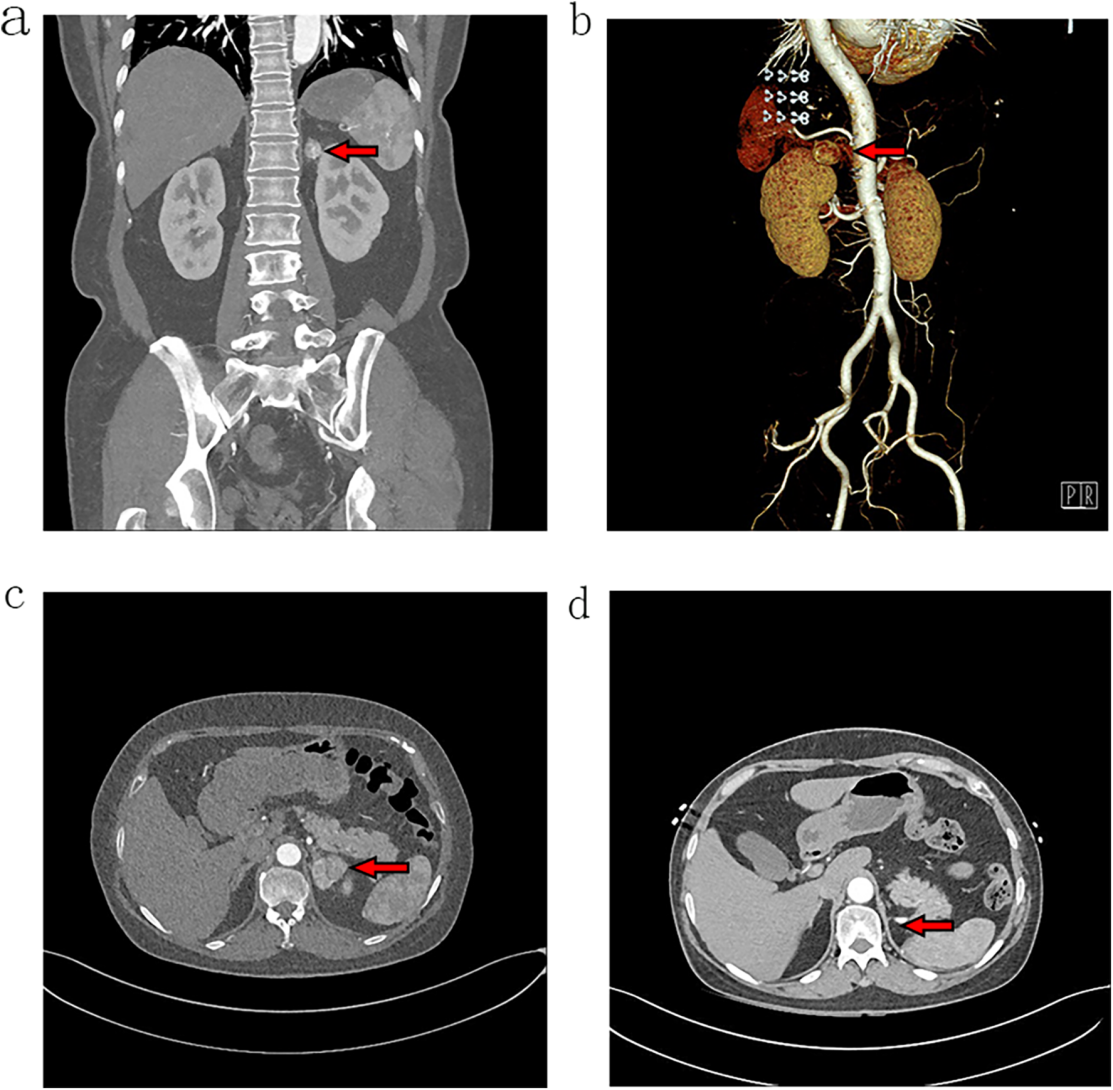
Abdominal CT images with contrast enhancement. Coronal views **(a,b)** and a cross-sectional view **(c)** reveal a soft tissue mass in the left adrenal region, characterized by mild and uneven enhancement. **(d)** The follow-up cross-sectional view at 6 months postoperatively hows no evidence of tumor recurrence in the left adrenal region.

A 48-year-old woman was treated in a tertiary hospital for “loss of consciousness.” The patient denied any symptoms related to upper abdomen discomfort, abdominal distension, hematuria, or fever. Physical examination revealed no tenderness in the upper abdomen or lumbar region, and no palpable abdominal masses were detected. An abdominal computed tomography (CT) scan identified a 24 × 31 mm soft tissue mass in the left adrenal region. A contrast-enhanced CT scan confirmed mild, uneven enhancement of the mass, with indistinct margins relative to the left adrenal gland. The bilateral kidneys were intact and showed average size, location, and enhancement. No mass was found in either kidney (Figures 1a–c). The plasma concentrations of adrenal-related hormones, including catecholamine, cortisol, renin, and aldosterone, were within normal ranges. Based on these findings, a preliminary diagnosis of a left adrenal mass was established. The differential diagnosis included adrenal cortical tumor, adrenal medullary tumor, extra-adrenal paraganglioma, and adrenal metastasis. The patient underwent laparoscopic surgery via a retroperitoneal approach to excise the mass. Intraoperatively, a 30 × 25 × 20 mm mass with an intact capsule was identified near the left adrenal gland. The mass was successfully resected outside the capsule, without involvement of the left kidney, spleen, or other adjacent organs.

**Figure 2:**
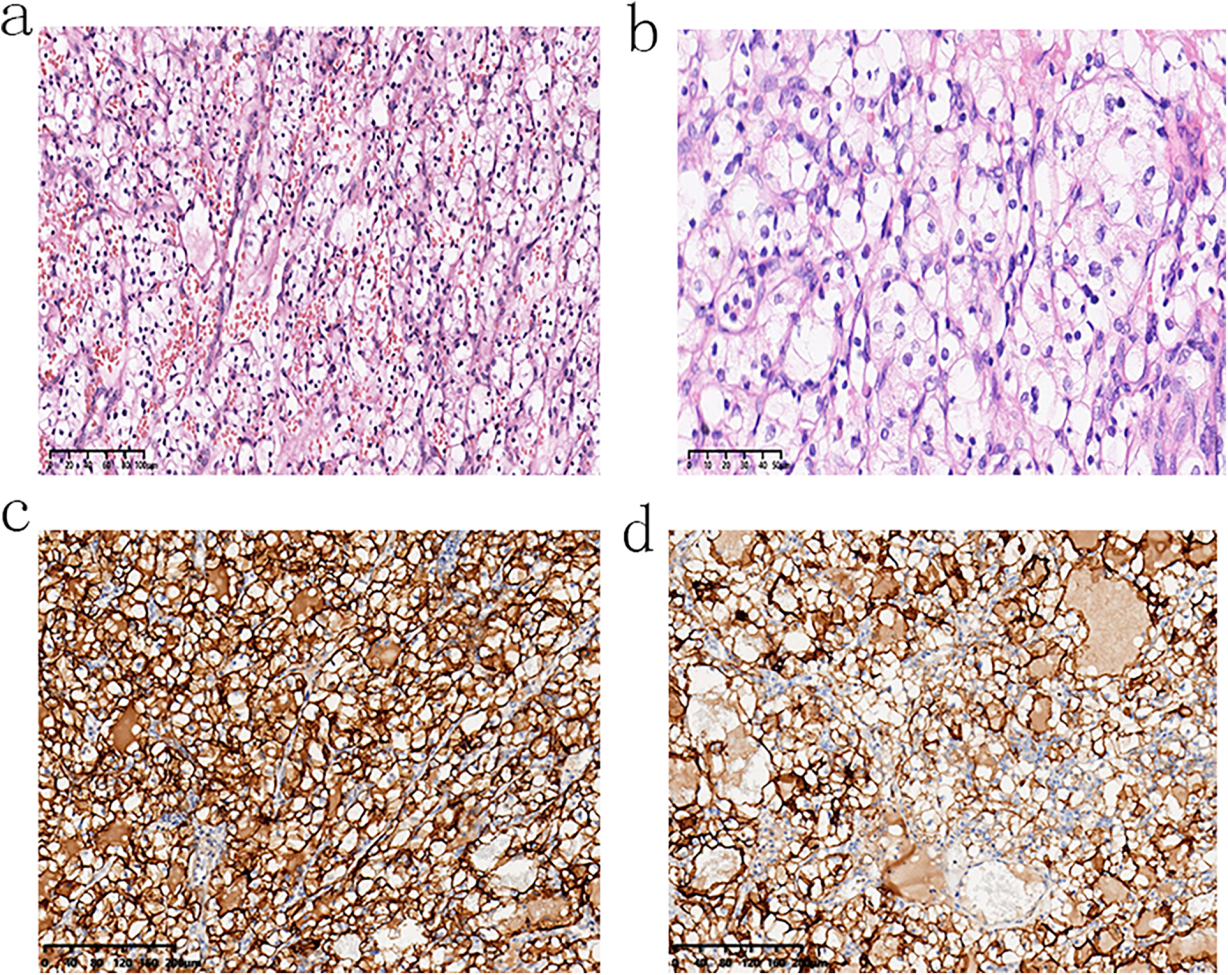
Microscopic examination of the tumor consistent with clear cell renal cell carcinoma: **(a)** at ×20 magnification and **(b)** at ×40 magnification. Immunohistochemical staining shows positive expression of **(c)** CA9 at ×40 magnification and **(d)** CD10 at ×40 magnification.

Histopathological examination revealed a well-encapsulated mass with a clear boundary from the surrounding tissues. Normal renal tissue was absent, but some normal adrenal tissue was observed in the surrounding area. Hematoxylin and eosin (H&E) staining showed tumor cells arranged in nest-like and adenoid structures, with a rich vascular network. The tumor cells were round to polygonal, with abundant clear cytoplasm and centrally located nucleus (Figures 2a,b). Immunohistochemistry results deemed the tumor cells positive for expression of Ki-67 (+, about 15%), PAX-8 (+), carbonic anhydrase 9 (CA9) (+), vimentin (foci +), CD10 (+) and negative for expression of markers such as chromogranin A (CgA) (-), melan-A (-), a-inhibin (-), calretinin (-), cytokeratin 7 (CK7) (-), RCC (-), and CD117 (-) (Figures 2c,d). These findings led to a pathological diagnosis of clear cell renal cell carcinoma (ccRCC), WHO/ISUP nuclear grade G2. There was no evidence of tumor involvement or invasion of the surrounding adrenal gland, and no normal renal tissue was found in the resected specimens.

The patient did not receive any adjuvant therapy postoperatively. At the 6-month follow-up, thoracoabdominal CT scans showed no signs of tumor recurrence or distant metastasis in the left adrenal area, thorax, or abdomen (Figure 1d). Written informed consent was obtained from the individual(s) for the publication of any potentially identifiable images or data included in this article.

## Discussion

Clear cell renal cell carcinoma (ccRCC) is the most common histological subtype of renal cell carcinoma (RCC). However, cases of extrarenal ccRCC are rare. Extrarenal ccRCC is believed to represent renal carcinoma arising from either remnants of embryonic kidney tissue or from an early developmental anomaly where kidney tissue remains in an extrarenal location (2).

Computed tomography (CT), magnetic resonance imaging (MRI), and contrast-enhanced ultrasound (CEUS) are commonly used in the diagnosis of renal and adrenal tumors. MRI offers high soft-tissue resolution and multi-parametric imaging, which is helpful in distinguishing benign from malignant tumors. However, the examination time is longer compared to other modalities. CEUS provides real-time dynamic observation of tumor blood perfusion, making it a simple and non-invasive option, but its field of view is limited and heavily dependent on the operator's technical skill. Preoperative biopsy can help determine the tumor's nature, but it carries risks such as damage to surrounding organs and tissues, potential bleeding, tumor seeding, and false-negative results. CT, on the other hand, remains the standard imaging modality for evaluating suspected renal and adrenal masses due to its fast scanning speed, multi-phase imaging capability, and broad applicability.

In the present case, CT imaging revealed a mass in the left adrenal region with mild, uneven enhancement and indistinct differentiation from the adrenal gland. The bilateral kidneys appeared normal in size, shape, and enhancement, making it challenging to distinguish this mass from primary adrenal tumors based solely on imaging. The imaging characteristics were similar to those seen in adrenocortical carcinoma. AlShalabi et al. reported a similar case where abdominal CT identified a large, irregularly enhanced tumor in the adrenal area, which was later confirmed as clear cell carcinoma following laparotomy and histopathological examination (5). Additionally, endoscopic ultra-sonography has been utilized in preoperative diagnosis of adrenal masses, though its use in extrarenal ccRCC remains limited (6).

The differential diagnosis for the mass included benign adrenal tumors, pheochromocytoma, adrenocortical carcinoma, metastatic carcinoma, and adrenocortical carcinoma. The absence of abnormal catecholamine, aldosterone, and cortisol levels, along with the lack of clinical symptoms associated with hormonal excess, effectively ruled out pheochromocytoma. Furthermore, the small size of the tumor, lack of local tissue invasion, and normal hormonal profile made adrenocortical carcinoma unlikely (7). No evidence of primary tumors elsewhere in the body was found on preoperative imaging, excluding metastatic carcinoma. Both kidneys were normal, and there was no anatomical or functional connection between the tumor and the left kidney.

Further, the tumor capsule was intact, which excluded the possibility of renal cancer invading the surrounding tissues or metastatic RCC. During the surgical procedure, the patient's kidneys on both sides remained undamaged and were not linked to the tumor. However, there was no obvious boundary with the adrenal gland. The tumor and a portion of the adrenal gland were completely excised. Pathological examination confirmed the diagnosis of extrarenal RCC, as no normal renal tissue was identified in the resected specimens.

Postoperative immunohistochemical analysis further supported the diagnosis of ccRCC, with positive staining for CA9, CD10, PAX-8, and vimentin, and negative staining for markers such as CgA, inhibin, melan-A, and calretinin. The negative staining for chromogranin A (CgA) ruled out pheochromocytoma, while the absence of melan-A, inhibin, and calretinin excluded adrenocortical tumors (8). The intact capsule and normal adrenal tissue adjacent to the mass indicated that the tumor had not infiltrated the adrenal gland.

The management of extrarenal ccRCC parallels that of typical ccRCC, with surgical resection being the primary treatment for localized tumors. In this case, the tumor was completely removed, and no recurrence was observed during the follow-up period. However, further studies are required to confirm the long-term efficacy of this approach. Robotic radiosurgery (RRS) has emerged as a non-invasive alternative to traditional renal mass surgery. Michael Staehler et al. demonstrated that RRS provided similar overall survival outcomes to open partial nephrectomy in elderly and high-risk patients (9).

Currently, there is no evidence to suggest that patients with locally advanced tumors benefit from regional or extended lymph node dissection, nor is there documented evidence supporting the efficacy of ipsilateral adrenalectomy in the management of locally advanced tumors. In fact, a large-scale study by the Mayo Clinic found no oncological benefit from routine ipsilateral adrenalectomy during radical nephrectomy for locally advanced renal cancer. The study also noted that ipsilateral adrenalectomy does not prevent contralateral adrenal metastasis, as the risk of metastasis is equivalent on both sides. Therefore, routine ipsilateral adrenalectomy is not recommended unless there is evidence of adrenal involvement or metastasis (10).

In summary, the treatment of advanced or metastatic extrarenal RCC remains reliant on systemic therapies, with palliative surgery or radiotherapy serving as adjuncts for managing primary or metastatic lesions. Diagnosing extrarenal ccRCC preoperatively, especially when tumors are located in the adrenal region, poses significant challenges. Future research should focus on optimizing the diagnostic and therapeutic strategies for this rare entity.

## Conclusion

This report presents a rare case of extrarenal clear cell renal cell carcinoma (ccRCC). The exact origin of extrarenal ccRCC remains unclear, potentially arising from embryonic remnants of renal tissue. Due to its rarity and the complexities of its development, extrarenal ccRCC poses diagnostic and therapeutic challenges. In accordance with established guidelines for renal cell carcinoma, complete surgical resection is the recommended treatment for localized extrarenal ccRCC. For cases that are advanced or metastatic, systemic therapy remains the cornerstone of management.

## Data Availability

The original contributions presented in the study are included in the article/Supplementary Material, further inquiries can be directed to the corresponding author.
